# Health Equity in Times of a Pandemic: A Plea for a Participatory Systems Approach in Public Health

**DOI:** 10.3389/fpubh.2021.689237

**Published:** 2021-10-29

**Authors:** Emelien Lauwerier, Sara Willems, Maïté Verloigne

**Affiliations:** ^1^Department of Experimental-Clinical and Health Psychology, Faculty of Psychology and Educational Sciences, Ghent University, Ghent, Belgium; ^2^Department of Public Health and Primary Care, Faculty of Medicine and Health Sciences, Ghent University, Ghent, Belgium

**Keywords:** health equity, systems thinking, participatory approach, public health, COVID-19

## Introduction

As we write, in early spring 2021, millions of people have been infected with COVID-19 and the majority of the population worldwide is still under some form of restricted movement. But vaccination campaigns are being set up and enrolled. Successful vaccination of the population is by all means the biggest key toward regaining normal life activities. It is now essential to make people to “behave as we want”—and to get them take the vaccine. In fact, all that has been expected from people during this pandemic—even for some time after successful vaccination—is “to behave in different ways”—be it to wash hands, wear face masks, stay at home, limit social contacts, and tele-work or have education from home. These universal preventive actions to fight the spread of COVID-19 thus depend for a great part on behavioral change.

## Social Structures Shape Our Life, and Choices

The idea is that when such behavior change is promoted, vulnerability to disease and severe outcomes is reduced significantly. All that must be done is increasing motivation to comply to those guidelines, and making people act upon their intentions. These premises rely on (social) psychology, explaining behavior change in terms of individual motives. Exemplary in this respect are intention, attitudes, self-efficacy, or outcome beliefs. During the past decades, various models have been developed that aim to explain human motivation in terms of a set of some of these cognitive constructs, such as the Health Belief Model (1), Protection Motivation Theory (2), Theory of Planned Behavior/Reasoned Action Approach (3–5), Social Cognitive Theory (6, 7), Health Action Process Approach (8), and implementation intentions (9). Although such theorizing is important and has informed effective interventions up to this point, they only reveal a minor part of the complex puzzle of behavior change. Behavior change is not easy for some people because of social determinants of health and health behavior (10). Examples include social structures such as social class, status, roles, groups, communities, and so forth. People who already live in poverty are hit harder by the pandemic crisis and by global measures that are installed as solutions to fight the crisis. Illiteracy, language barriers, poor working and living environments, lack of access to care, and lack of information are only a few of many barriers faced by poor communities leading to inefficient and untimely responses to the pandemic. Also, poorer communities have less power and agency, which are needed to co-define solutions for their problems (11). This co-definition is key to the process of empowerment and may help to regain power and control, and change social and political environments leading to mastery, improved equity, and better quality of life in the long term (12). We therefore need to step away from global actions as the sole solutions to health crises and healthcare in general for disadvantaged populations and move toward participation of these communities in the public health debate. This solution requires a different frame, one that is able to understand the already existing differences in (determinants of) health and interconnectedness between social environmental levels. A system lens or approach is suited to deal with this complexity, and in what follows we first discuss its principles and key elements. Next, we accentuate participatory actions as a method for bringing about change in a complex social environment.

## Systems Thinking and Public Health: What Is It, and Why Adopting It?

A systems approach relates to the ideas of complexity theory, complexity science, or models of living systems (13, 14) and outlined below are its main distinctive characteristics:

- A systems approach is “holistic” and considers the fact that multiple influences impact behavior change, such as in case of a compliance with COVID-19 measures. This idea of multiple influences is also incorporated in so-called ecological models on health behavior, differentiating between the individual level (e.g., knowledge, beliefs, and lifestyle), the micro level (e.g., family, households), the meso level (e.g., communities), and the macro level (e.g., policies, rules, and sanctions). A systems level goes further in that it also articulates interactions within and between the different layers. Applied to the COVID-19 global pandemic response, we should recognize the following: policies and universal actions (developed on a macro level) are implemented in (and transformed through) communities (meso level) interacting with (and transformed through) other meso-structures such as local health organizations and schools residing into (and transformed once again by) families and other social interactions (micro level), impacting the individual. It is therefore easy to see why global actions may not have their intended impact on the individual, as the input of those global measures are filtered, changed, and transformed through and within the different layers surrounding the individual.
- All layers should also be considered as being a system itself. The systems themselves also evolve in an organic way, which means in an unexpected, non-trivial, complicated way. Agents or actors within those systems interact, adapt their behavior based on feedback and this living nature makes it highly unpredictable and difficult to alter (11–13).
- The complexity within systems thinking also relates to the presence of non-linear outcomes: what first may act as an input or trigger may become an output at a later stage (13–15). Referring to the COVID-19 pandemic: social distancing may reduce infection rate, and this may increase self-confidence and hopes about the future. These outcomes may become essential conditions for people to comply with other measures, leading to better (mental) health outcomes on the long term. In communities where lockdown is burdensome and hard to accomplish, these intermediate outcomes may not easily be attained, pertaining to already existing health inequities.

These distinctive characteristics call for another paradigm or approach that distinguishes itself from the traditional population-based or top-down approach. At its core, systems thinking requires a consideration of human behavior in terms of how humans interact with each other in networks (16). This paper is not about how methodologies and operational methods may be aligned to the complexity of systems thinking, but about one of the most crucial aspects with important implications for academia, health professionals, and policies: being the necessity to transcend conventional boundaries and act in a participatory way with the communities at stake. The COVID-19 crisis acts as a magnifier revealing the lack of preparedness to support and treat disadvantaged populations illustrated by a sole and inappropriate top-down response treating all groups equally. At the same time, however, it also made us double aware of numerous local initiatives set in place that invested into working co-productively with disadvantaged groups in society and having impact, albeit on a much smaller scale. In what follows, we briefly discuss participatory methods and principles, and translate these to the current pandemic crisis meaning co-production with and empowerment of disadvantaged groups.

## A Participatory Approach: A New Frontier in Public Health?

There are a number of participatory methodologies or participatory paradigms, including Participatory Action Research (17, 18), Community-Based Participatory Research (19, 20), and Participatory Health Research (21). They all share the same key principle of participation of the target group (i.e., the people within the community) and other relevant actors to co-produce knowledge. Very importantly, a true participatory approach implies that the people within the community are at least equitably involved and share power and responsibilities (21). Participation is not a dichotomous concept but is conceived to vary along a continuum from low to high control. However, when taking a hierarchical approach to define participation (22), real decision-making power from the community members is needed to be able to call it “participatory.”

Important prerequisites of true levels of participation are methods that allow to start where the people are: to build and foster a strong partnership with stakeholders and trustful relationships with the people in the community in order to facilitate the open dialogue between all parties involved (19, 21). With regard to the COVID-19 situation, preventive actions should thus ideally be developed and implemented in close collaborations with people for whom the actions are meant and with the stakeholders from all environmental levels. Other researchers have also already underlined the importance of such participatory approach in a pandemic (23, 24). This will ensure actions are tailored to the context of the community and solutions to achieving positive changes are developed locally (25). Moreover, as a result of participation, there is more social cohesion within the community and people within the community are empowered through co-learning and experience feelings of ownership (26, 27). These participatory outcomes (independent from the solutions that have been co-created) can also impact people's health and well-being (27). Nevertheless, it is important to acknowledge that using a participatory approach requires considerable time and resources and an active commitment from all parties involved (17). Attached to this article is a powerful example of a local initiative [i.e., VZW Zuidpoort from the city of Ghent in Flanders (Belgium), see Appendix 1 in the Supplementary Material] that aligns with a system view and uses key principles of participation with the groups in society they engage with. The example offers an excellent learning opportunity and in the next section we will draw upon our theoretical descriptions and case illustrations and delineate key lessons that have to be learnt about how to better support vulnerable communities during health crises and more efficiently address their health needs in general. It will hopefully trigger readers to make or continue plans that allow for more durable and targeted health actions for those in highest need.

## Summarizing Notes and Challenges for the Future

Reflecting on the current COVID-19 situation from a systems point of view and keeping participatory work in mind, we may make a few summarizing notes as lessons to be learnt that may optimize our public health response to health crises and health promotion in general for vulnerable communities. Also, some challenges remain and must be tackled, and we address a few below.

First, in a crisis, measures to prevent a rapid spread of the virus need to be taken urgently by policy makers. Therefore, taking global, top-down actions to induce behavioral change in all people instead of a more considerate approach seems at that specific moment the easiest thing to do. One could argue that there is too little time to tailor these global actions, as this requires good and detailed insights into the needs and characteristics of specific communities. However, it could also be considered, even during crisis, to pass on some power or even equal power to local stakeholders that have already insights into these needs and characteristics because of their longstanding tradition of investing in strong partnerships and trustful relationships in more vulnerable communities (such as illustrated within the case example). There is clear evidence supporting this claim coming from the combat against other deadly viruses, such as the Ebola virus in Western Africa. In the beginning of the Ebola response, suspicion against the motives of global actions to reduce transmission of the virus appeared to be high, and hindered implementation of control and safety measures (28–30). It was only later that one recognized that community engagement was not a barrier but the major key in combatting the virus. Strategies such as investment in trusted local leaders, communication through trusted channels and resources, and decentralized actions that allow for flexibility and adaptation to local needs were among the important lessons learnt within that context. While context differs, those community engagement strategies may very well be a better response to the COVID-19 pandemic compared to a global response. A targeted approach may seem more considerate but induce higher effects, even shortly after putting it in place.

Second, from a systems point of view, public health interventions are more successfully embedded if these are co-produced with stakeholders for whom the intervention should make a difference (25). Within a community, this means co-production with for instance local policy makers, organizational representatives, citizens, and so forth. When measures have to be introduced quickly, such as in case of the COVID-19 pandemic, co-design is challenging, and highly unlikely. However, at the minimal, measures can always be checked with representatives of the community and even the slightest changes may make a difference. If there are local stakeholders that have strong links with the community, that have experience with participation of the community, and whom—above all—community members trust, this would support and accelerate the process (29).

Third, local resources and people might also be important chains in terms of monitoring the COVID-19 situation and deliver more specific data on for instance virus outbreaks, vaccination readiness and behavior, etc. This is challenging, as evaluation and monitoring is often considered to be the scope of grand, non-locally organized instances (for instance in Belgium, this would constitute the Flemish governmental policy level). Also, communities may lack capacities and resources to efficiently monitor public health actions, suggesting training, and educating local professionals in health and social care in undertaking monitoring and evaluation may be needed. Public health is not only a matter of “having the right numbers” but also of “empowering those at stake in taking control of their situation,” which is also in line with participatory principles. Knowing what is at stake and being able to track progress through measurable indicators is an important step in taking control.

Fourth and relatedly, a participatory approach implies freedom of communities based on local knowledge and wisdom (19). However, this is challenging. Very often during this pandemic crisis, we noticed disturbances between global action and local necessities. In Belgium, most of the official measures in the beginning of the pandemic were on hygiene and social restrictions, leaving those from poorer communities particularly behind. Often, they do not have the same access to information to be able to adhere to these measures, making them more vulnerable. They also tend to have bigger families and higher social support needs. Lockdown measures make them therefore also disproportionally more vulnerable for isolation and exhaustion, and poor mental health in the longer term. There is no easy solution here but key is to shift to more balanced public health policies that also allow for bottom-up, local strategies involving participation of communities in highest need. One of the first steps probably is to be more prepared that different groups need different approaches to reach them, by using for example different channels and networks through which to convey messages, and to allow for transformation of those messages to the particularities of specific groups.

In conclusion, participation is a core method of power-giving and building capacity, and essential in giving voice to communities that are sometimes left unheard. If a context and if the issue allows, participation may very well be the best answer in establishing equitable and healthy societies. Crisis, such as the COVID-19 pandemic, may justify more passive approaches, such as providing information and consultation, to avert disaster. We however proposed some ways of increasing participation, even during health crises, including the excellent example of VZW Zuidpoort Gent. We used this crisis as a magnifier and want to draw attention to the continued need for high-level participatory actions, being true participation, and empowerment, that are installed in a durable, non-fragmented way.

## Author Contributions

EL and MV wrote the paper. All authors participated in the concept of the paper and reviewed the manuscript.

## Conflict of Interest

The authors declare that the research was conducted in the absence of any commercial or financial relationships that could be construed as a potential conflict of interest.

## Publisher's Note

All claims expressed in this article are solely those of the authors and do not necessarily represent those of their affiliated organizations, or those of the publisher, the editors and the reviewers. Any product that may be evaluated in this article, or claim that may be made by its manufacturer, is not guaranteed or endorsed by the publisher.
