# Supplementary material for: Health Equity in Times of a Pandemic: A Plea for a Participatory Systems Approach in Public Health
Source: Front Public Health. 2021 Oct 29;9:689237. doi: 10.3389/fpubh.2021.689237 (PMC8586000; doi:10.3389/fpubh.2021.689237)
Supplement: Supplementary file 1 [file Data_Sheet_1.PDF]

Appendix 1: Case study VZW Zuidpoort

General description

VZW Zuidpoort is “an association in which people in poverty take the floor”, located in a deprived neighborhood in the city of Ghent, Belgium. The mission of the association is (a) to be a safe meeting place for all people in the community, and (b) to fight against poverty and social inclusion together with those at stake. They are an important partner for sensitization, influencing policies and poverty reduction.

The organization: a “system” of multiple actors and participatory action at its base

Participation is one of the main pillars of the association. VZW Zuidpoort always starts from the perspectives, the experiences and the stories of the people in poverty themselves, and encourage others to do as well. By using this participatory approach, the voices of the people at stake are heard and they feel empowered in their struggle against poverty. Also within the organization, the active involvement of employees, volunteers, but also members in (managing) the organization is stimulated. VZW Zuidpoort strongly emphasizes that poverty is not a choice, and that it is maintained by how the society is organized. Therefore, they as an organization (meso level) do not only focus on individuals and families/households (micro level) within the community (meso level), but also work together with other organizations (meso level) and local policy makers (macro level) in order to target the different influential layers. Importantly, they stimulate and initiate (participatory) interaction between actors from these layers, which can lead to adjustments within a specific layer. A simple example from within the association itself is that they organize a “parliament” on a monthly basis in which the people from the community can comment and give input on the activities and initiatives in order to further adapt and improve the operation of VZW Zuidpoort. Another example is that VZW Zuidpoort regularly invites local policy makers to listen to the stories of the people in the community in order to make adjustments within the policy system by taken the perspectives of people in poverty into account when developing and implementing local policy measures.

Illustration of a prepared response to health crisis: the organization during the COVID-19 pandemic

With regard to the COVID-19 pandemic, this association has taken and still is taking initiatives to **support people in poverty with regard to the population-based, global actions,** all with a participatory mindset. An important facilitating factor here is that VZW Zuidpoort had already the tradition to collaborate with people in poverty from the community and with important

actors. As a result, the organization had already good insights into the needs and characteristics of these people. To make it more specific, we provide some examples of their COVID-19 initiatives, which should be seen as a chain of continuing interactions and efforts to support those people. Although the examples are specifically related to COVID-19, they are also relevant to provide a general overview on how public health responses to future crisis situations might be improved.

- A lot of emphasis was put on clearly communicating about the global, population-based strategies to minimize the spread of the virus. After each federal press conference in Belgium, the organization developed infographics (in different languages) to explain the new measures in an accessible way. In addition, their overview prioritized measures relevant for people in poverty, e.g. measures related to public transport were more important than measures related to travelling outside of Europe. After developing more than 20 infographics, the city of Ghent has then taken on the social task to develop and spread new infographics for each press conference.
- When the measures were very strict, VZW Zuidpoort organized activities that were still allowed in order to improve people's feelings of well-being (e.g. weekly walks with a limited amount of people), offered leisure-time activities through their website that could be done from home (i.e. following a theater show) and gave people the opportunity to reserve the garden of the association (especially for those not having an outer place). Organizing these activities as an immediate action in a crisis-situation naturally requires a good and thorough understanding of the needs of people in poverty.
- Based on the input from people in poverty, VZW Zuidpoort in collaboration with other organizations has provided a clear overview of guidelines (e.g. related to communication, education, services, social contacts, etcetera) for the local policy makers and societal actors on which there should be a focus in times of a pandemic. These guidelines are a strong signal on how to deal with the aftermath of COVID-19 in the next upcoming years, but can also be useful in a broader sense than COVID-19.
- Together with or supported by other organizations, VZW Zuidpoort has collaborated with the city of Ghent and the Flemish Agency for Care and Health to give feedback and further improve the vaccination letter for citizens before

being actually send out, to make sure it is readable and understandable for all people.
